# Targeting mTOR signaling overcomes acquired resistance to combined BRAF and MEK inhibition in *BRAF*-mutant melanoma

**DOI:** 10.1038/s41388-021-01911-5

**Published:** 2021-07-24

**Authors:** Beike Wang, Wei Zhang, Gao Zhang, Lawrence Kwong, Hezhe Lu, Jiufeng Tan, Norah Sadek, Min Xiao, Jie Zhang, Marilyne Labrie, Sergio Randell, Aurelie Beroard, Eric Sugarman, Vito W. Rebecca, Zhi Wei, Yiling Lu, Gordon B. Mills, Jeffrey Field, Jessie Villanueva, Xiaowei Xu, Meenhard Herlyn, Wei Guo

**Affiliations:** 1 Department of Biology, School of Arts & Sciences, University of Pennsylvania, Philadelphia, PA USA; 2 Molecular and Cellular Oncogenesis Program and Melanoma Research Center, The Wistar Institute, Philadelphia, PA USA; 3 Department of Translation Molecular Pathology, University of Texas MD Anderson Cancer Center, Houston, TX USA; 4 Center for Systems Biology, Lunenfeld-Tanenbaum Research Institute, Mount Sinai Hospital, Toronto, ON Canada; 5 Department of Computer Science, New Jersey Institute of Technology, Newark, NJ USA; 6 Department of Cell, Developmental and Cancer Biology, School of Medicine, and Knight Cancer Institute, Oregon Health & Science University, Portland, OR USA; 7 Department of Genomic Medicine, Division of Cancer Medicine, The University of Texas MD Anderson Cancer Center, Houston, TX USA; 8 Department of Systems Pharmacology and Translational Therapeutics, Perelman School of Medicine, University of Pennsylvania, Philadelphia, PA USA; 9 Department of Pathology and Laboratory Medicine, Perelman School of Medicine, University of Pennsylvania, Philadelphia, PA USA; 10 Present Address: Department of Neurosurgery, The Preston Robert Tisch Brain Tumor Center and Department of Pathology, Duke University Medical Center, Durham, NC USA

**Keywords:** Cancer therapeutic resistance, Melanoma

## Abstract

Targeting MAPK pathway using a combination of BRAF and MEK inhibitors is an efficient strategy to treat melanoma harboring *BRAF*-mutation. The development of acquired resistance is inevitable due to the signaling pathway rewiring. Combining western blotting, immunohistochemistry, and reverse phase protein array (RPPA), we aim to understanding the role of the mTORC1 signaling pathway, a center node of intracellular signaling network, in mediating drug resistance of *BRAF*-mutant melanoma to the combination of BRAF inhibitor (BRAFi) and MEK inhibitor (MEKi) therapy. The mTORC1 signaling pathway is initially suppressed by BRAFi and MEKi combination in melanoma but rebounds overtime after tumors acquire resistance to the combination therapy (CR) as assayed in cultured cells and PDX models. In vitro experiments showed that a subset of CR melanoma cells was sensitive to mTORC1 inhibition. The mTOR inhibitors, rapamycin and NVP-BEZ235, induced cell cycle arrest and apoptosis in CR cell lines. As a proof-of-principle, we demonstrated that rapamycin and NVP-BEZ235 treatment reduced tumor growth in CR xenograft models. Mechanistically, AKT or ERK contributes to the activation of mTORC1 in CR cells, depending on *PTEN* status of these cells. Our study reveals that mTOR activation is essential for drug resistance of melanoma to MAPK inhibitors, and provides insight into the rewiring of the signaling networks in CR melanoma.

## Introduction

Metastatic melanoma is the most aggressive form of skin cancer [1]. Approximately 50% of cutaneous melanomas harbor activating *BRAF* mutations, which drive hyperactivation of the Mitogen-Activated Protein Kinase (MAPK) signaling pathway [2, 3]. Targeting MAPK pathway using a combination of BRAF and MEK inhibitors elicits a 70% response rate in patients with *BRAF*-mutant melanoma [4–6]. Additionally, adjuvant use of BRAF/MEK inhibitors resulted in a lower risk of relapse in patients with stage III melanoma [4]. Despite the encouraging observations, the development of acquired resistance is almost inevitable, limiting the efficacy and duration of these targeted therapies. Dynamic rewiring of signaling networks allows tumor cells to adapt to the BRAF and MEK inhibitors treatment. Molecular mechanisms that underlie acquired resistance to BRAF inhibitor monotherapy can be attributed to the reactivation of ERK, activating mutations in *NRAS*, alternative activation of RTK-mediated pathways, amplification or truncation of *BRAF*, overexpression of *COT*, mutations in *MEK1* and other genetic events [5–10]. In terms of resistance to BRAFi and MEKi combination therapy (CR), several mechanisms including the development of *MEK2* mutations, acquisition of concurrent *BRAF/NRAS* mutations, amplification of *BRAF*, ER translocation of the ERK and others have been reported [11–16]. In addition, MAPK-independent mechanisms such as the rewiring of RAC1/CDC42-PAK signaling pathway [17], alternative activation of the PI3K/AKT signaling axis [15], and immune-related components in tumor microenvironments also contribute to CR [18].

mTOR is a conserved serine and threonine protein kinase that plays a critical role in cell growth by regulating transcription, protein synthesis, ribosome biogenesis, and cell metabolism [19–24]. mTOR kinase acts in two functionally distinct complexes, mTOR complex 1 (mTORC1) and 2 (mTORC2), whose activities and substrate specificities are regulated by their co-factors [25–27]. Aberrant mTOR activation has been observed in many types of cancers including metastatic melanoma [28–33]. mTORC1 and mTORC2 activity is regulated by the PI3K pathway in response to growth factors and cell stress stimuli [34–38]. In addition, activation of ERK also leads to increased mTOR activity in mTORC1 through TSC2 phosphorylation [39]. Since both the MAPK/ERK and PI3K/AKT signaling pathways converge into mTORC1, this signaling pathway may be crucial for the efficacy of targeted therapy in patients with *BRAF*-mutant melanoma. Decreased mTORC1 activity following MAPK inhibition has been shown to be necessary for the induction of apoptosis and cell cycle arrest in melanoma cells [40]. Resistance to BRAF or MEK inhibitors is also associated with the adaptive induction or persistence of the activation of the AKT pathway [41, 42], an upstream regulator of mTORC1. Resistance could also be reversed by inhibiting PI3K [43–45].

In this study, we have systematically studied mTORC1 signaling in different subsets of established *BRAF*-mutant melanoma cells that acquired resistance to combined BRAF and MEK inhibition. We show that mTORC1 reactivation plays a pivotal role in development of CR. The activation of mTORC1 requires ERK or AKT, depending on *PTEN* status of the tumors. We further show that the recovery of mTORC1 activity represents a therapeutic vulnerability for CR *BRAF*-mutant melanoma. mTOR inhibitors significantly inhibited proliferation and induced apoptosis of CR cells. Our study provides a molecular mechanism by which *BRAF*-mutant melanoma cells gain resistance to BRAFi and MEKi combination therapy, and implicates mTOR inhibition for further treatment of CR patients.

## Results

### mTORC1 activity is restored in melanoma cells resistant to BRAFi/MEKi combination

**Fig. 1:**
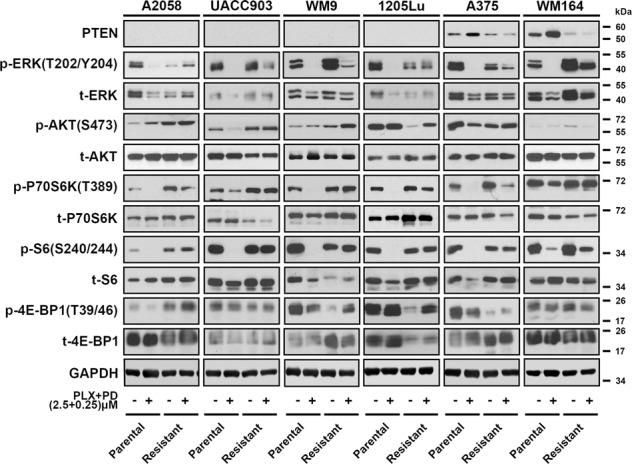
Recovery of mTORC1 activity in CR melanoma cell lines. Western blot analysis showing the phosphorylation levels of proteins associated with mTORC1 and AKT signaling pathways in parental and CR melanoma cells. Data are representative of three independent biological experiments.

To better understand the effect of combined BRAFi and MEKi on resistance-associated mTORC1 signaling, we treated a panel of paired parental and CR *BRAF* mutant melanoma cell lines with PLX-4720 (PLX, 2.5 μM, BRAFi) and PD0325901 (PD, 0.25 μM, MEKi) for 24 h (Fig. 1). Of these cell lines, A2058, UACC903, and WM9 are *BRAF* mutant cells with homozygous *PTEN* loss; 1205Lu has heterozygous *PTEN* loss; A375 and WM164 are cell lines with wild-type *PTEN* [46–49]. Our assay confirmed the loss of *PTEN* in A2058, UACC903, and WM9, and expression in A375 and WM164 melanoma cells. PTEN was barely detectable in 1205Lu cells, despite its heterozygous loss of *PTEN*. Expression of p-ERK (T202/Y204) was decreased in parental cells with the combined BRAF and MEK inhibition, but was unchanged or only slightly decreased in CR melanoma cell lines after the same treatment. The BRAFi and MEKi combination markedly reduced p-S6^S240/244^ and p-P70-S6K^T389^ levels in parental cells, but recovered in CR cells. Since both p-S6^S240/244^ and p-P70-S6K^T389^ are direct downstream components of mTORC1 signaling, these results suggest that mTORC1 signaling was initially suppressed by combined BRAF and MEK inhibition, but later rebounded upon gaining resistance. Phosphorylation of another mTORC1 downstream molecule, 4EBP1, did not appear to be inhibited by the combined BRAFi and MEKi in either parental or CR cells. This may be attributed to the complicated array of upstream regulators for 4EBP1 such as p53, PI3K, and ATM [50]. Additionally, phosphorylation of AKT was increased in the CR cells with *PTEN* loss, suggesting that adaptive induction of PI3K/AKT signaling pathway could contribute to the survival of CR cells. We noted that, in parental cells with wild-type *PTEN*, combined BRAF and MEK inhibition increased the expression of PTEN. Similar observation was reported in melanoma cells treated with MEK inhibitor [51].

### mTORC1 activity in CR melanoma xenograft and PDX models

**Fig. 2:**
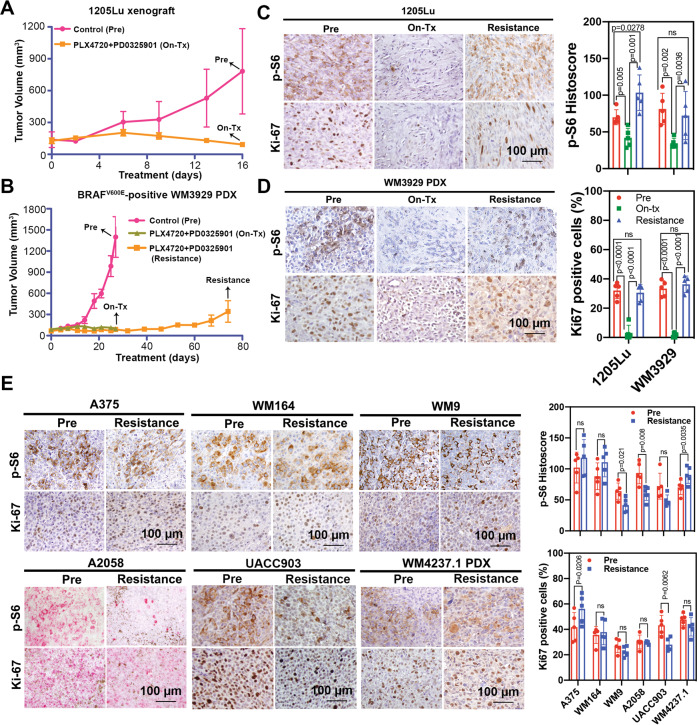
Restoration of mTORC1 activity in CR melanoma xenografts and PDXs. Tumor growth curve of 1205Lu xenografts (**A**) and WM3929 PDX (**B**) before treatment (“Pre”), with short-term treatment (“On-Tx”) and relapsed after long-term treatment (“Resistance”) with combined BRAF and MEK inhibitors, *n* = 5 mice for each indicated group. Black arrows represent the time points of sample collection. Representative IHC (left) and quantification (right) of p-S6 and Ki-67 staining of the 1205Lu xenograft (**C**) and WM3929 PDX (**D**) tumors. The *H*-score of IHC images for each mouse (*n* = 5 mice per group) were quantified from five high-power fields. **E** Representative IHC images (left) and quantification (right) of p-S6 and Ki-67 staining in the melanoma xenograft tumors. Data are presented as mean ± SD. *P* values are based on a two-sided unpaired Student’s *t*-test.

Next, we examined mTORC1 signaling in xenografts of paired parental and CR melanoma cell lines as well as patient-derived xenograft models. To test our hypothesis that mTORC1 activity was initially suppressed during short-term MAPKi and rebounded after long-term treatment, we examined 1205Lu xenografts and WM3929 PDX tumors that were untreated (denoted as “Pre”), short-term treated with BRAF and MEK inhibitors (denoted as “On-tx”), or a long-term treated until resistance arose (denoted as “Resistance”). Tumor growth was substantially inhibited upon the short-term treatment with the BRAF and MEK inhibitor combination (On-tx group), and the tumors gradually progressed after 60 days of treatment (Resistance group), suggesting CR was successfully established in these cells (Fig. 2A, B). To determine if mTORC1 activity was restored in CR tumors, we examined the levels of S6 phosphorylation, and Ki67 as a marker of proliferation. We found that p-S6^S240/244^ and Ki67 levels were inhibited in both 1205Lu xenografts and WM3929-PDX tumors during the short-term treatment but restored in the CR xenografts (Fig. 2C, D), indicating that the mTORC1 signaling is reactivated in CR melanoma in vivo. We asked whether there was any difference in baseline level of p-S6 between parental and CR xenografts. Immunohistochemistry (IHC) staining of p-S6 ^S240/244^ and Ki67 was performed in paired A375, WM164, WM9, A2058, UACC903 parental and CR xenografts and WM4237.1 parental and CR PDX (Fig. 2E). Ki67 expression was not obviously different in four out of six of the paired parental and CR xenografts, suggesting that CR xenografts have similar proliferative ability as their parental counterpart. The levels of p-S6 in A375-CR, WM164-CR, UACC903-CR, and WM4237.1 CR-PDX xenografts were comparable to their parental counterparts, suggesting mTORC1 signaling in CR xenografts recovered to baseline levels after development of resistance.

### mTOR inhibition induces cell cycle arrest and inhibits CR cell proliferation

**Fig. 3:**
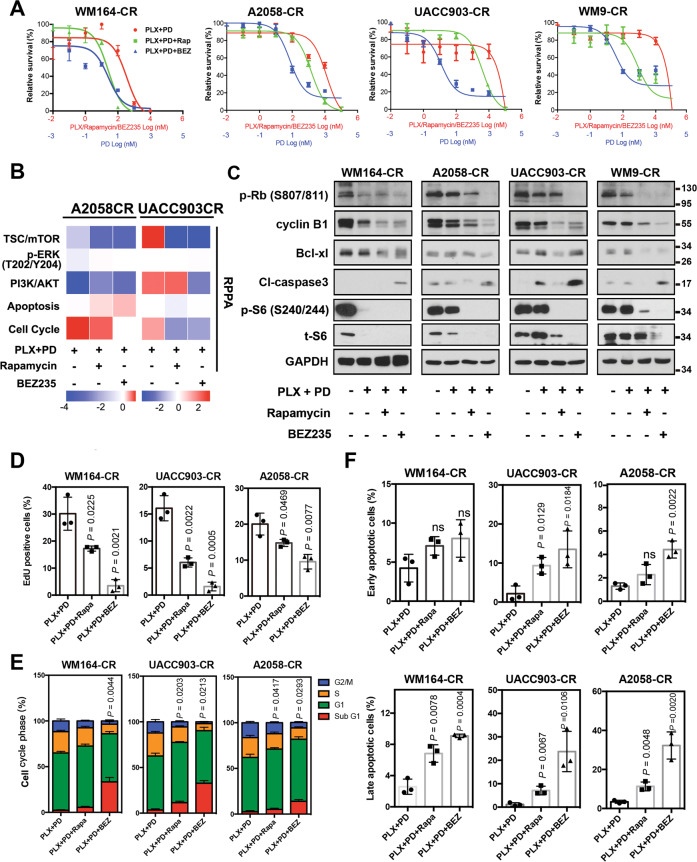
mTOR inhibitors induce cell cycle arrest and apoptosis in CR melanoma cells. **A** Relative survival of WM164-CR, A2058-CR, UACC903-CR, and WM9-CR cells treated with increasing concentrations of Rapamycin (Rapa, Max = 100 µM), NVP-BEZ235 (BEZ, Max = 100 µM), PLX4720 (PLX, Max = 100 µM) and PD0325901 (PD, Max = 10 µM) for 72 h. **B** Heatmaps of indicated pathway scores from RPPA analysis of A2058-CR and UACC903-CR cells after treatment with PLX (2.5 µM) + PD (0.25 µM) in the absence or presence of Rapa (100 nM) and BEZ (1 µM) for 24 h. **C** The expression of cell cycle and apoptosis -related proteins was analyzed by western blotting in CR cells treated with indicated inhibitors (PLX, 2.5 µM; PD, 0.25 µM; Rapa, 100 nM; BEZ, 1 µM) for 72 h. **D** Quantification of EdU incorporation assays performed to detect the proliferation of WM164-CR, UACC903-CR, and A2058-CR melanoma cells treated with Rapamycin (Rapa, 100 nM) and NVP-BEZ235 (BEZ, 1 µM) in the presence of PLX (2.5 µM) and PD (0.25 µM) for 24 h. **E** Flow cytometry analysis of cell cycle stages of cells in (**D**). **F** Quantification of early and late apoptotic cell population treated with Rapamycin and NVP-BEZ235 in the presence of BRAF and MEK inhibitors by the Annexin V/7-AAD double staining. Data are presented as mean ± SD (*n* = 3). *P* values (vs. PLX + PD treatment group) are from a two-sided unpaired Student’s *t*-test.

To test whether inhibition of mTORC1 could overcome resistance to combined BRAF and MEK inhibition, we used rapamycin, an allosteric mTORC1 inhibitor. We also included in our experiments NVP-BEZ235 (BEZ235), a dual PI3K and mTOR inhibitor [52]. For cell viability detection, both rapamycin and BEZ235 sensitized CR cells to combined BRAF and MEK inhibition (Fig. 3A, Figs. S1 and S2A, B). BEZ235 alone showed inhibitory effect on the growth of CR cells, and the inhibitory effect was further enhanced in most cells when combined with BRAF and MEK inhibitors. Rapamycin alone did not show obvious inhibitory effects (Fig. S1). We next performed reverse phase protein array (RPPA) on two of the cell lines, A2058-CR and UACC903-CR (Fig. 3B). The TSC/mTOR signaling pathway and cell cycle progression were downregulated in both A2058-CR and UACC903-CR cell lines, accompanied by increased apoptosis. Western blotting analysis showed that rapamycin decreased the levels of p-P70-S6K^T389^ and p-S6^S240/244^ in CR cells (Fig. 3C and Fig. S3). As a dual PI3K-mTOR inhibitor, BEZ235 also inhibited the phosphorylation of AKT in all *PTEN* loss CR cells, whereas rapamycin increased the p-AKT level in A2058-CR cells and did not change the p-AKT level in UACC903-CR and WM9-CR cells (Fig. S3). BEZ235 also inhibited the phosphorylation of P70-S6K, S6, and 4E-BP1 in CR cell lines, consistent with the inhibition of both mTORC1 and PI3K. Both rapamycin and NVP-BEZ235 downregulated cell cycle-related proteins such as p-Rb and cyclin B1 and the anti-apoptotic protein Bcl-xL in these CR cell lines (Fig. 3C). Rapamycin induced moderate apoptosis in some cells, whereas BEZ235 induced much stronger apoptosis. Similar observation was made in a previous study demonstrating that rapamycin combined with PI3K inhibitor could induce marked cell death in the *de novo* resistant melanoma cell lines to PLX4720 by blocking AKT phosphorylation [53]. We further analyzed the effect of mTOR inhibition on proliferation rate, cell cycle progression, and induction of apoptosis in these CR cells. Rapamycin significantly reduced the percentage of EdU-positive cells in WM164-CR (Rapa vs. Control: 17.24 ± 2.20% vs. 30.14 ± 11.8%, *P* < 0.05), UACC903-CR (Rapa vs. Control: 6.10 ± 2.581% vs. 16.25 ± 7.1%, *P* < 0.05) and A2058-CR (Rapa vs. Control: 12.21 ± 2.870% vs. 19.21 ± 6.0%, *P* < 0.05) cells, while BEZ235 almost completely inhibited cell proliferation in WM164-CR, UACC903-CR and A2058-CR cells (Fig. 3D and Fig. S2C). Flow cytometry analysis showed that rapamycin and BEZ235 led to an increase of CR cells in G_1_ phase. In addition, BEZ235 led to an increase in the percentage of cells in sub-G_1_ phase, consistent with BEZ235 inducing apoptosis in CR melanoma cells (*P* < 0.01 in WM164-CR and A2058-CR, *P* < 0.05 in UACC903-CR) (Fig. 3E). We also analyzed the effect of combination treatment using Annexin-V/7-AAD double staining. Rapamycin led to a moderate induction of apoptosis, while BEZ235 induced apoptosis in these three CR melanoma cell lines (*P* < 0.01 in WM164-CR and A2058-CR, *P* < 0.05 in UACC903-CR) (Fig. 3F and Fig. S2D). This is consistent with a previous study showing that MEK1/2 and a PI3K/mTOR inhibitor was more effective in the activation of Bax and caspase-3 and in the induction of caspase-dependent apoptosis [54].

**Fig. 4:**
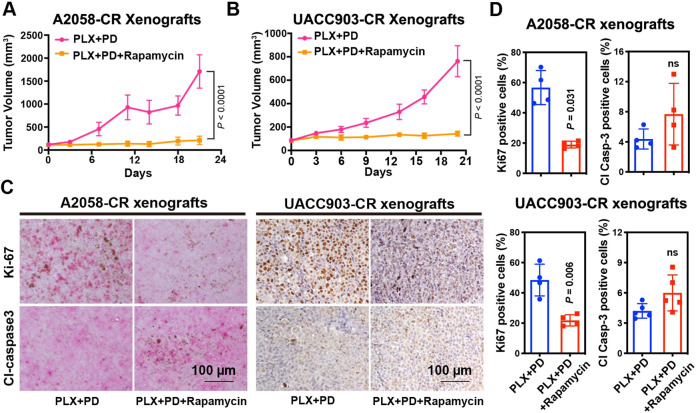
Rapamycin suppresses the growth of CR melanoma xenograft tumors. Tumor growth curve of A2058-CR xenografts (**A**) and UACC903-CR xenografts (**B**) treated with rapamycin in the presence of PLX4720 and PD0325901, *n* = 5 mice for each indicated group. **C** Representative Ki67 and cleaved caspase-3 IHC images in the xenografts treated with vehicle or rapamycin in the presence of PLX4720 and PD0325901. **D** Quantification of Ki67 and cleaved caspase-3 in the tumors. The indicated molecular of positive expression level for each mouse (*n* = 4 mice per group) were quantified from 5 high-power fields. Data are presented as mean ± SD. *P* values are from a two-sided unpaired Student’s *t*-test.

To evaluate the effect of mTOR inhibitors in vivo, we treated A2058-CR and UACC903-CR xenograft tumors with BRAF and MEK inhibitors together with rapamycin or BEZ235. The addition of rapamycin or BEZ235 inhibited tumor growth for both A2058-CR and UACC903-CR xenografts (Fig. 4A, B; Figs. S4 and S5A, B). Analysis of the treated tumors by IHC staining showed a substantial decrease in the percentage of Ki67 positive cells in both A2058-CR and UACC903-CR tumors after combined treatment with rapamycin, PLX-4720, and PD0325901 (Fig. 4C, D). However, consistent with the in vitro data, the percentage of cleaved caspase-3 positive cells were not significantly changed, suggesting that rapamycin did not elicit obvious apoptosis in CR xenografts. Taken together, our results provide a rationale to inhibit mTORC1 activity to overcome acquired resistance to combined BRAF/MEK inhibitors.

### Contribution of PI3K/AKT and ERK to mTORC1 activity in CR cells

**Fig. 5:**
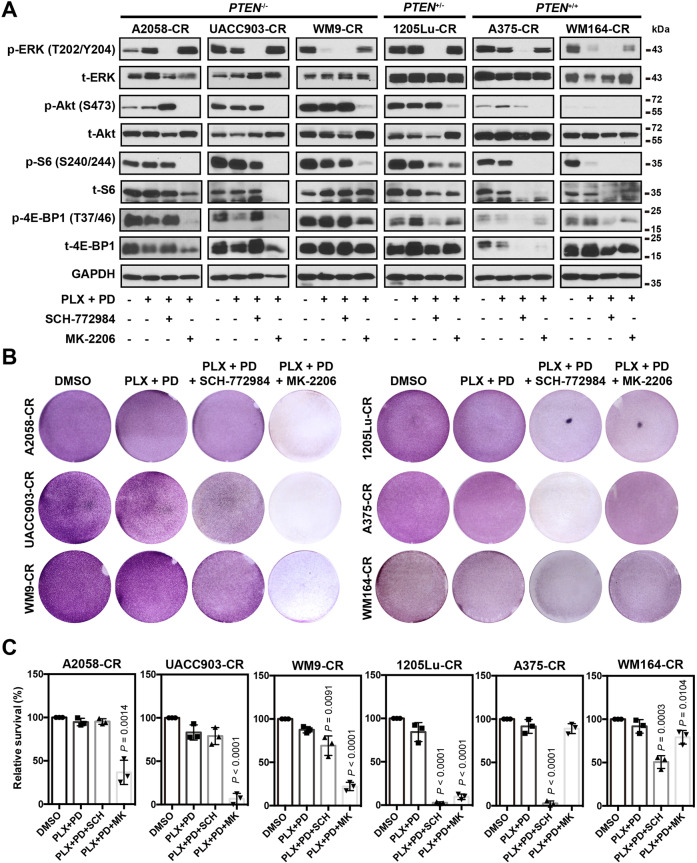
mTORC1 activity in CR melanoma cells is regulated by ERK and AKT pathway. **A** The activation status of mTORC1 pathway in melanoma CR cells with indicated treatment. SCH-772984 and MK-2206 were used to inhibit the activation of ERK and AKT, respectively. *PTEN*^−/−^ represents cell lines with homozygous *PTEN* loss; *PTEN*^+/−^ for cells with heterozygous *PTEN* loss; *PTEN*^+/+^ for cells with wild-type *PTEN*. **B** Crystal violet assay of CR cells treated with SCH-772984 or MK-2206 in the presence of PLX-4720 and PD0325901. **C** Quantification of the crystal violet staining in (**B)**. Data are presented as mean ± SD (*n* = 3). *P* values (vs. PLX + PD treatment group) are from a two-sided unpaired Student’s *t*-test.

**Fig. 6:**
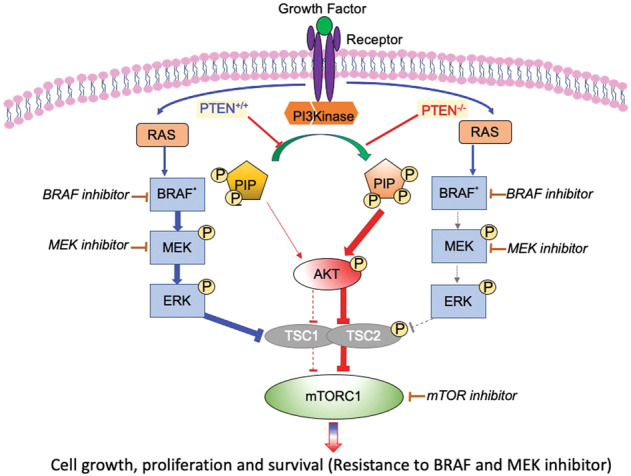
Schematic diagram showing the contribution of MAPK signaling and PI3K/AKT signaling in CR melanoma cells. For CR melanoma with wild-type *PTEN*, MAPK signaling plays an important role in mTORC1 activity restoration. For CR melanoma with *PTEN* loss, mTOR signaling restoration relies on the PI3K/AKT activation. See text for details.

We next sought to understand the molecular mechanism underlying the recovery of mTORC1 activity in melanoma cells in the context of acquired resistance to the BRAFi and MEKi combination. Since MAPK and AKT signaling pathways stimulate mTORC1 activity, we treated CR cells with either the ERK inhibitor, SCH-772984, or the AKT inhibitor, MK-2206, in addition to the BRAFi and MEKi combination. In cell lines with complete *PTEN* loss (A2058-CR, UACC903-CR, and WM9-CR), SCH-772984 did not downregulate p-S6^S240/244^ or p-4E-BP1^T37/46^, suggesting that the activity of mTORC1 in these CR cells is independent on the MAPK pathway (Fig. 5A). In contrast, MK-2206 significantly suppressed the expression of p-S6^S240/244^ and p-4E-BP1^T37/46^ in these cell lines, suggesting that mTORC1 activity was dependent on AKT activity. In *PTEN*^*+/−*^ or *PTEN*^*+/+*^ cells (1205Lu-CR, A375-CR, and WM164-CR cells), however, both SCH-772984 and MK-2206 suppressed the levels of p-S6 and p-4E-BP1 (Fig. 5A), suggesting that mTOR signaling in these cell lines was regulated by both AKT and ERK signaling. Next, we examined whether inhibition of AKT or ERK changes the growth of CR melanoma cells. MK-2206 significantly impaired the viability of the *PTEN*^*−/−*^ cells (A2058-CR, UACC903-CR, and WM9-CR), while the ERK inhibitor had a limited effect (Fig. 5B, C). Both AKT and ERK inhibitors significantly decreased the viability of 1205Lu-CR (*PTEN*^*+/−*^), while ERK inhibitors were more effective for WM164-CR and A375-CR cells (*PTEN*^*+/+*^). These results suggest that the differential sensitivity of CR cells to AKT vs. ERK inhibition depends on their *PTEN* status. Based on our results, a model illustrating the different contributions of MAPK and PI3K/AKT to mTOR-mediated CR is presented in Fig. 6.

## Discussion

The major challenge of targeted therapy for treating metastatic melanoma is acquired drug resistance [7, 9, 55–57]. Our previous study underscored the importance of signaling network rewiring in the acquisition of drug resistance to combined BRAF and MEK inhibitors in metastatic melanoma [17]. In the current study, we focused on mTORC1 because it is a nexus targeted by upstream signals such as PI3K pathway and MAPK pathway [58]. Recently, several mTOR inhibitors have been approved by the U.S. Food and Drug Administration to treat different types of cancers [59, 60]. However, whether and how mTORC1 activation contributes to acquired resistance of combined BRAF and MEK inhibition, and whether mTOR inhibitors have a therapeutic benefit through overcoming combined therapy resistance remain elusive.

Previous studies have examined the role of mTOR signaling in naive melanoma cell lines with *de novo* resistance or acquired resistance to BRAFi or MEKi [9, 40, 51, 53, 61, 62]. Taking advantage of an array of CR cell lines and xenografts, we now present evidence for a crucial role of mTORC1 in *BRAF*-mutant CR melanoma. We observed the restoration of mTORC1 activity in CR cells. In addition to BRAF mutant melanoma, enhanced mTORC1 activity has also been reported to associate with acquired resistance to combined inhibition of CDK4/6 and MEK in *NRAS*-mutant melanomas [63].

Previous studies suggested that co-targeting the AKT/mTOR pathway reversed the resistance of some melanoma cell lines with intrinsic cross-resistance to BRAF and MEK inhibitors [43, 44, 64]. These studies suggest that, regardless of the mechanism of resistance (i.e. MAPK activation or AKT activation), addition of mTOR inhibitor could be an effective way to restore their sensitivity to BRAF or MEK inhibitors. Our results provide evidence that mTOR inhibitor in combination with BRAF and MEK inhibitors blocked the growth of CR melanoma cells primarily due to cell cycle arrest. Targeting eIF4F and S6, two critical mTORC1 downstream proteins, has also been shown to be an effective approach to overcoming drug resistance of melanoma to MAPK inhibitors [63, 65, 66]. Moreover, in a previous clinical study, dual inhibition of mTOR and MAPK signaling pathways doubled the progression-free survival benefit relative to monotherapy (ClinicalTrials.gov identifier: NCT01390818). A recent Phase I clinical study also showed that the combination of BRAF inhibitor (vemurafenib) with rapamycin (everolimus, rapamycin analogs) is safe and well tolerated, with partial responses among different cancers, including patients who previously experienced progression on BRAF and/or MEK inhibitor therapy [67]. However, it was also reported that inhibition of mTORC1 by rapamycin and its analogues resulted in hyperactivation of AKT through the release of S6K1 and IRS-1 negative feedback loop [68]. This could be a concern for the use rapamycin. Deng et al. reported rapamycin needed to be combined with PI3K inhibitor to induce more cell death in *de novo* resistant cell lines to BRAFi [53]. Shi et al. demonstrated that PI3K and mTORC1/2 inhibition should be combined with BRAF or MEK inhibitor for higher inhibitory response in BRAFi resistant cell lines because rapamycin caused compensatory survival signaling even with simultaneous inhibition of MEK and/or AKT [9]. Gopal et al. also showed that inhibition of AKT, mTOR1/2, or insulin-like growth factor I receptor resulted in higher synergistic cell killing of melanoma cell with *de novo* resistance to MEKi than rapamycin [51]. On the other hand, while dual PI3K and mTOR inhibitor showed improved efficacy in inhibiting tumor growth in preclinical models, adverse effects and poor tolerability were also reported for pancreatic neuroendocrine tumors patients [69–73].

To determine the molecular mechanism for the restoration of mTORC1 activity, we evaluated the activation status of ERK and PI3K/AKT pathways in CR melanoma cells. Reactivation of the MAPK pathway has been proposed to be a major mechanism underlying the resistance of melanoma cells to BRAFi [74], and the combination of BRAF and MEK inhibition [75]. Previous studies have demonstrated that resistant melanoma cells displayed activated ERK due to a *MEK2* mutation and *BRAF* amplification, which contributed to sustained mTOR activity in resistant cells [61, 62]. Our data suggest that ERK activation was important for the recovery of mTORC1 activity only in a subset of CR melanoma cell lines that express wild-type *PTEN*, where the RAS/ERK pathway is a major regulator of mTORC1. On the other hand, AKT inhibition decreased mTORC1 activity in all the CR melanoma cells lines tested, suggesting that AKT is upstream of mTORC1 activity in cells with mutant or wild-type *PTEN*. These results are consistent with the findings that the activation of PI3K/AKT pathway is crucial in the acquisition of drug resistance in melanoma [74, 76, 77]. Silva et al. reported that MAPK inhibition was able to inhibit mTORC1 activity in treatment-naive *BRAF*-mutant melanoma cells, while AKT inhibition failed to suppress this activity [78]. Based on our results, we propose that activation of mTORC1 activity shifts from ERK to the PI3K/AKT pathway in CR melanoma cells, and that upstream activation of mTORC1 activity in these melanoma cells is closely associated with *PTEN* status. These data are in agreement with the earlier reports demonstrating *PTEN* genetic status was associated with intrinsic and acquired drug resistance in melanoma cells [76, 79, 80]. We show that mTOR inhibition is generally effective in decreasing the survival of CR cells, with CR cells with *PTEN* loss having higher sensitivity to mTOR inhibitor (Fig. 3F; Fig. S2A, B). Previous studies showed receptor tyrosine kinase (RTK) upregulation and activation contributed to the resistance to BRAFi or MEKi [10, 42, 51], which may also involve the restoration of mTORC1 activity through the PI3K/AKT and MAPK pathways. However, the exact mechanism of RTKs for mTOR activation in different CR melanoma situation needs further in-depth study.

In summary, our study demonstrates that the restoration of mTORC1 activity is important in mediating acquired resistance of *BRAF*-mutant melanoma to MAPK inhibitors. While future studies are needed to understand the upstream signaling events such as RTK upregulation and *NRAS* mutations in mTOR activation, our study warrants further evaluation of PI3K/mTOR inhibitors to overcome resistance to combined BRAF/MEK inhibitors. Furthermore, *PTEN* status should be considered in CR melanoma patients when selecting appropriate second line therapies.

## Materials and methods

### Cell lines, reagents, and antibodies

Human metastatic melanoma cell lines were established at the Wistar Institute as previously described [17]. They were authenticated by DNA fingerprinting and were tested regularly before assays to avoid mycoplasma contamination. Melanoma cells were cultured in RPMI 1640 (Invitrogen) supplemented with 5% FBS (Gibco). The resistant cells that acquired resistance to PLX-4720 (10 µM) and PD0325901 (1 µM) combination were established after continuous treatment, and the CR cells were maintained with the combination of PLX-4720 at 2.5 μM and PD0325901 at 0.25 μM throughout the experiments. PLX-4720. SCH-772984 and MK-2206 were purchased from Selleck, Inc. PD0325901, rapamycin (Rapa), NVP-BEZ235 (BEZ) were purchased from LC Laboratories. All information about the primary antibodies is included in Table S1. Secondary antibodies were purchased from Cell Signaling Technology (CST).

### Cell viability, proliferation, cell cycle, and apoptosis assays

Cell viability was assayed by crystal violet staining. Briefly, equal amounts of cells were seeded in six-well plates at 60% confluence overnight and then treated with DMSO or specific inhibitors (PLX + PD: 2.5 + 0.25 µM; Rapamycin (Rapa): 100 nM; NVP-BEZ235 (BEZ): 1 µM; SCH-772984: 2 μM and MK-2206: 1 μM) for 3 days. The cells were then washed with PBS and fixed with glutaraldehyde solution (500 µl glutardialdehyde in 12.5 ml PBS, pH 7.4) for 20 min. After rinsing with PBS, cells were stained with 0.02% crystal violet solution for 30 min. After extensive washing with distilled water, cells were air-dried.

Cell number and growth were measured by MTT assays using the Cell Proliferation Kit I (Roche) according to the manufacturer’s instruction. The cells were seeded into the 96-well plate at a density of 5000 cells/well overnight, followed by treatment with serial dilution of drugs for 72 h. The maximum concentration for the indicated drugs as followed: Rapa, 100 µM; BEZ, 100 µM; PLX-4720 (PLX, 100 µM) and PD0325901 (PD, 10 µM). The dilution ratio is 1:10. At least three independent experiments were performed. The IC50 values were calculated from dose-response curves using GraphPad Prism 6.0.

For 5-ethynyl-2′-deoxyuridine (EdU) cell proliferation assay (Ribo-Bio Co., Ltd., Guangzhou, China), CR cells were seeded on cover slips at a density of 1 × 10^5^ cells/ml overnight. The attached cells were processed for the indicated treatments for 24 h. All the inhibitors used in the treatments are listed as followed: Rapa, 100 nM; BEZ, 1 µM; PLX, 2.5 µM and PD, 0.25 µM. 2 h before collection, 100 μL EdU solution (50 μM) was added into the medium. The collected cells were fixed in 4% paraformaldehyde for 20 min and subsequently permeabilized with PBS containing 0.5% Triton X-100 for 20 min. The cells were then stained with 100 µL 1×Apollo^®^ stain liquid at room temperature away from light for 30 min, followed by DAPI staining for 5 min. After washing with PBS, the cells were mounted and examined under a fluorescence microscope. The percentage of EdU-positive cells was calculated from five random fields in three wells.

Apoptosis was determined by Annexin-V APC/7-Aminoactinomycin D (7-AAD) double-staining. After treated with Rapa, 100 nM, BEZ, 1 µM in the presence of PLX, 2.5 µM and PD, 0.25 µM for 72 h, CR cells were collected and washed twice with cold cell staining buffer (Biolegend, San Diego, CA). Then 5 µl FITC Annexin-V staining solution (Biolegend) were mixed with cells at a concentration of 1 × 10^6^ cells/ml, and kept in dark on ice for 15 min. 5 µl 7-AAD solution was added and incubated at room temperature for 5 min. Subsequently, with 400 µl Annexin V binding buffer added to each cell suspension, the cells were immediately analyzed using a BD LSRII flow cytometer, the results of which were analyzed using FlowJo software (TreeStar Inc.).

For cell cycle analysis, after treatment with Rapa, 100 nM, BEZ, 1 µM in the presence of PLX, 2.5 µM and PD, 0.25 µM for 24 h, CR cells were harvested and fixed with ice-cold 70% ethanol for at least 2 h at 4 °C. Ethanol was added dropwise to the cell pellet while vertexing. Cells were then treated with DNase-free RNase (200 μg/ml) for at least 2 h. After centrifugation, cells were resuspended in propidium iodide solution (20 μg/ml) for 30 min at room temperature in dark and subsequently analyzed using a FACS Calibur. The analysis for cell cycle was performed as previously described [17].

### Western blot analysis

Cell lysates were prepared in RIPA buffer supplemented with protease and phosphatase inhibitor cocktail (Roche) 24 h after the different drug treatments. After centrifugation at 16,000 *g* for 30 min at 4 °C, protein concentration was determined by Bradford assays (Bio-Rad). Equal amounts of protein were loaded and separated by sodium dodecyl sulfate polyacrylamide gel electrophoresis, and transferred onto nitrocellulose membranes. After blocking with 5% nonfat milk at room temperature for 1 h, the diluted primary antibody was utilized to incubate with the nitrocellulose membranes at 4 °C overnight. The immunoactivity was detected with horseradish peroxidase-conjugated secondary antibodies (anti-mouse and anti-rabbit, both from CST). Subsequently, the blots were developed with ECL Western Blotting Substrate (Pierce). Information about the primary antibody is listed in Table S1.

### Immunohistochemistry (IHC)

IHC was performed on formalin-fixed, paraffin-embedded sections. Briefly, after deparaffinization, antigens were retrieved by steaming the tissue section in citrate buffer (10 mmol/L, pH 6.0) and Tris-EDTA buffer (pH 9.0) for 5 min. After blocking with Protein Block Serum-Free (Dako, Agilent), sections were incubated with the primary antibody overnight at 4 °C. Following incubation with a biotinylated secondary antibody (1:200) for 30 min, the peroxidase activity of sections was visualized with Fast Red or DAB (Vector) and counter-stained with Hematoxylin (Life technologies). Five areas containing the highest numbers of stained cells within each section were selected for histologic quantification under the light microscopy at ×400 magnification. For calculating *H*-score, a semiquantitative approach was used as previously described [81–83]. The pathologist who examined the tumor sections was blinded to the treatment information. All the primary antibody information has been listed in Table S1.

### Patient specimen collection and generation of patient-derived xenograft (PDX)

Clinical data and tissue collection from patients with Stage IV melanoma was performed with informed consent at the University of Pennsylvania Abramson Cancer Center in accordance with the Institutional Review Board (Protocol number 707906). PDX tumors derived from patients with metastatic melanoma who progressed on the combined targeted therapies were expanded in vivo using NOD/SCID/IL-2Rγ^−/−^ (NSG) mice before the therapy experiments as previously described [84, 85]. The expansion phase was under continuous drug pressure at approximately clinical plasma levels.

### Animal studies

All animal experiments were reviewed and approved by Institutional Animal Care and Use Committee (IACUC) of the University of Pennsylvania and the Wistar Institute. For the establishment of drug-resistant melanoma xenograft models, a suspension of A2058-CR or UACC903-CR cells (5 × 10^6^ cells/100 μl) was inoculated into the flanks of 8-week old female athymic nu/nu mice. Each group has 5 mice, which is determined based on the studies in the similar field. Tumor size was measured by a digital caliper and the volume was calculated by the formula: length × (width)^2^/2. When the tumor volume reached ~100 mm^3^, mice were then allocated randomly to different treatment groups. All the CR xenografts had been fed with chow containing Dabrafenib (150 mg/kg) and Trametinib (1.5 mg/kg) (Bio-Serv). Mice bearing the established A2058-CR or UACC903-CR xenograft were treated with PLX-4720 (200 p.p.m.) and PD0325901 (7 p.p.m.) individually or in combination with rapamycin (12.5 mg/kg) by oral gavage twice daily. For the control group, the matching xenografts were administrated with an equal amount of vehicle. Mice were euthanized before the tumor reached 10% of the body weight or the longest tumor dimension reached 2.0 cm. The health status of the mice was monitored during the course of the experiment in case of signs of distress. The mice were euthanized once determined to have poor health condition score according to IACUC policy. No specific blinding technique was used for the animal studies.

### RPPA

The analysis of RPPA data was performed according to the protocol from the M.D. Anderson Cancer Center [86]. Specifically, relative protein levels for each sample were determined by interpolation of each dilution curves from the “standard curve” (supercurve) of the slide (antibody). Supercurve is constructed by a script in R written by the RPPA core facility. These values are defined as Supercurve Log2 value. All the data points were normalized for protein loading and transformed to linear value, designated as “Normalized Linear”, which was transformed to Log2 value, and then median-centered for further analysis. Median-Centered values were centered by 31 subtracting the median of all samples in a given protein. All of the above-mentioned procedures were performed by the RPPA core facility. Pathway scores were calculated according to a previous publication [87]. The pathway predictors used to calculate each pathway score had been listed in Table S2. The normalized data provided were visualized using MORPHEUS (https://software.broadinstitute.org/morpheus/).

### Statistical analysis

Statistical analyses were performed using GraphPad Prism 6.0. Sample sizes were determined based on the studies we and other researchers published. Normality of distribution was determined by D’Agostino-Pearson omnibus normality test and the equal variance assumption among groups was assessed by Brown–Forsythe test. One-way ANOVA was used to compare mouse tumor volume data among different groups. Two-sided Student’s *t*-tests were used for pair-wise comparison of the remaining datasets. Data are represented as mean ± SD. A two-tailed value of *P* < 0.05 was considered statistically significant.

## Supplementary information

Analysis of CR cell growth using MTT assay.

mTOR inhibitors suppress the viability of CR cells via decreasing proliferation and inducing apoptosis.

Activation of AKT/mTOR signaling pathway in CR melanoma cells treated with Rapamycin or NVP-BEZ235.

Spider blots of the CR melanoma xenograft tumor growth when treated with rapamycin or NVP-BEZ235 in the presence of BRAF and MEK inhibitors.

NVP-BEZ235 suppresses the growth of CR melanoma tumors.

Supplementary Table and Supplementary Figure Legends
