# Supplementary figures and images for: Targeting mTOR signaling overcomes acquired resistance to combined BRAF and MEK inhibition in BRAF-mutant melanoma
Source: Oncogene. 2021 Jul 24;40(37):5590–9. doi: 10.1038/s41388-021-01911-5 (PMC8445818; doi:10.1038/s41388-021-01911-5)

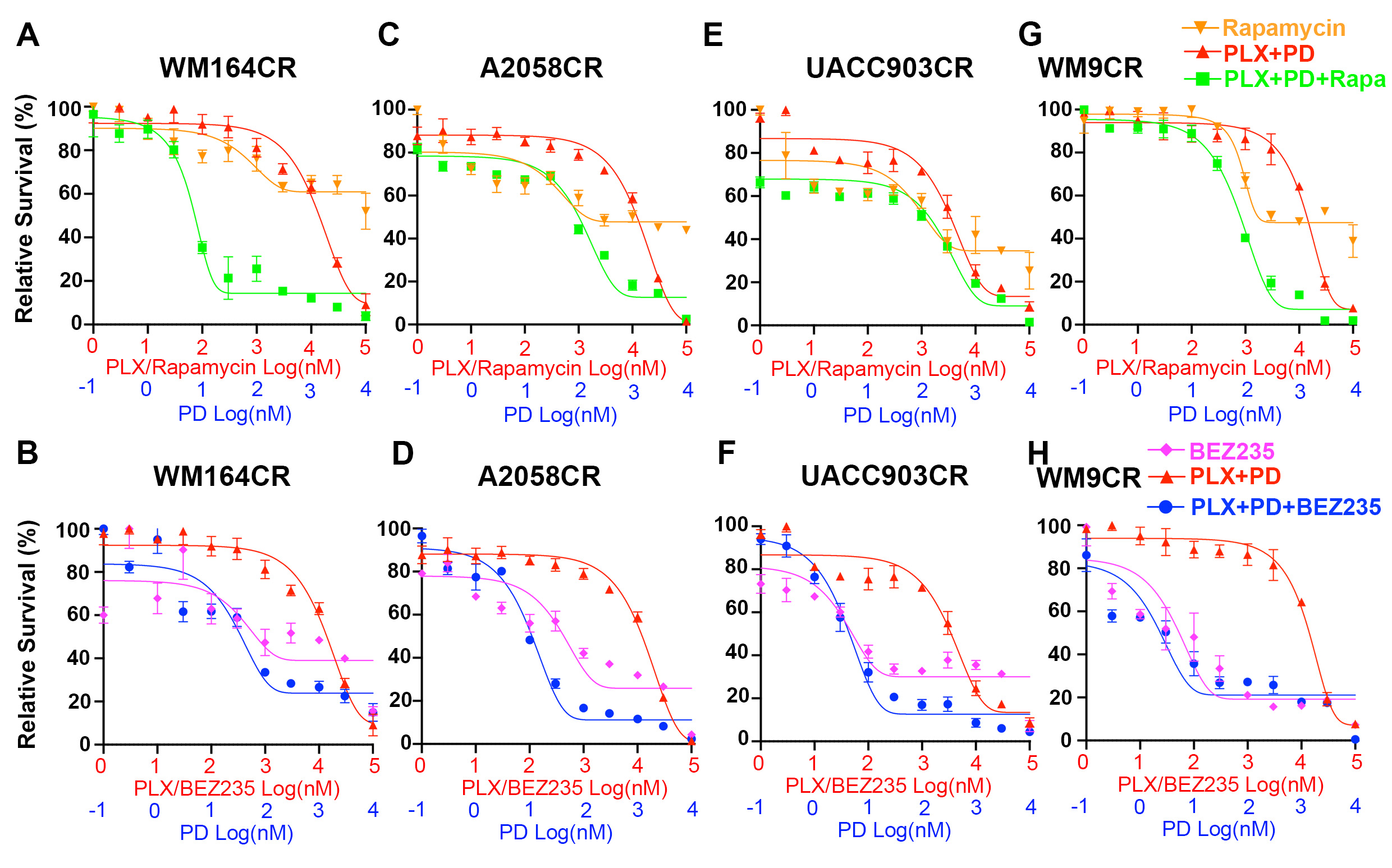

Supplement: Supplementary file 1 — Analysis of CR cell growth using MTT assay. [file 41388_2021_1911_MOESM1_ESM.jpg]

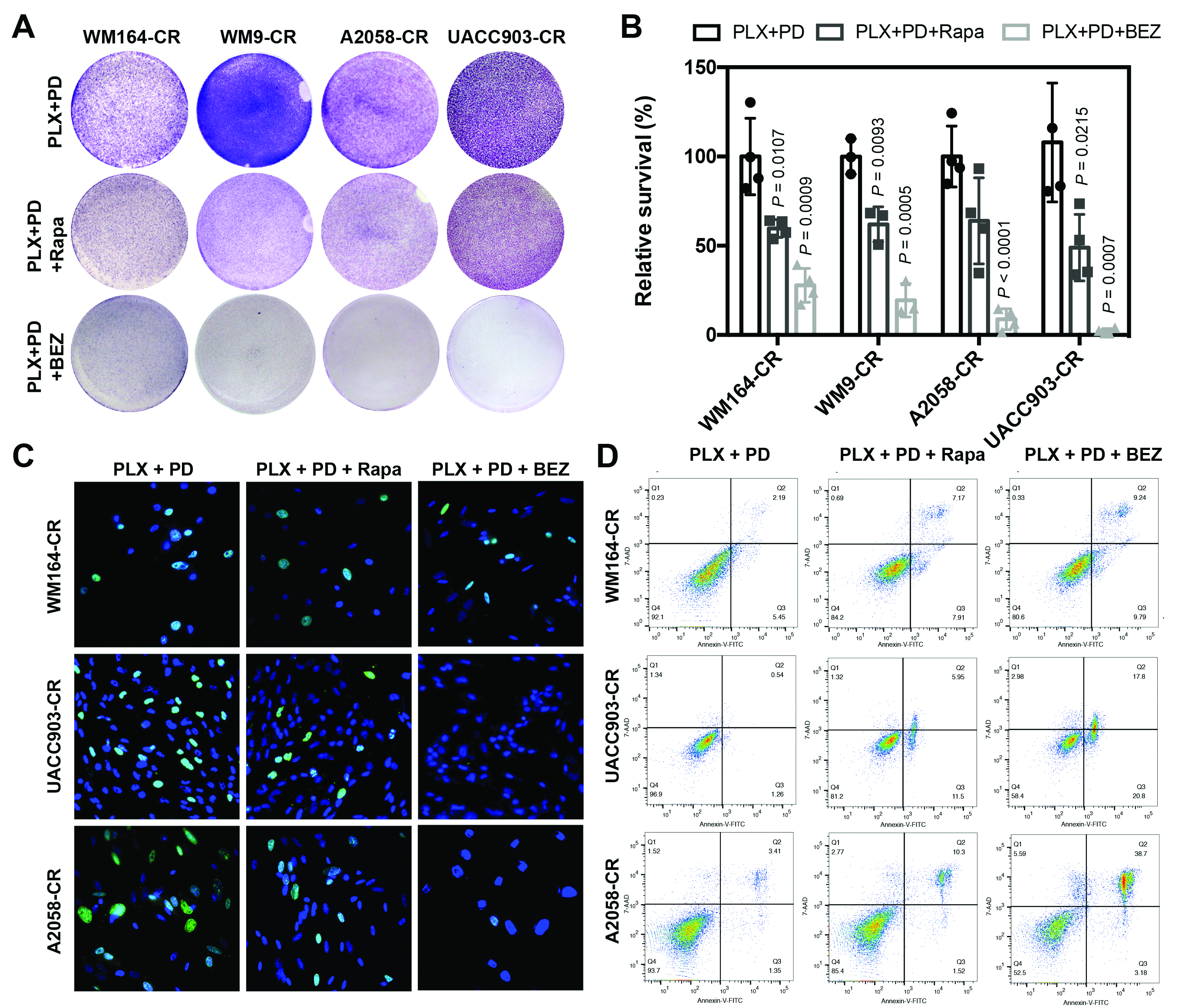

Supplement: Supplementary file 2 — mTOR inhibitors suppress the viability of CR cells via decreasing proliferation and inducing apoptosis. [file 41388_2021_1911_MOESM2_ESM.jpg]

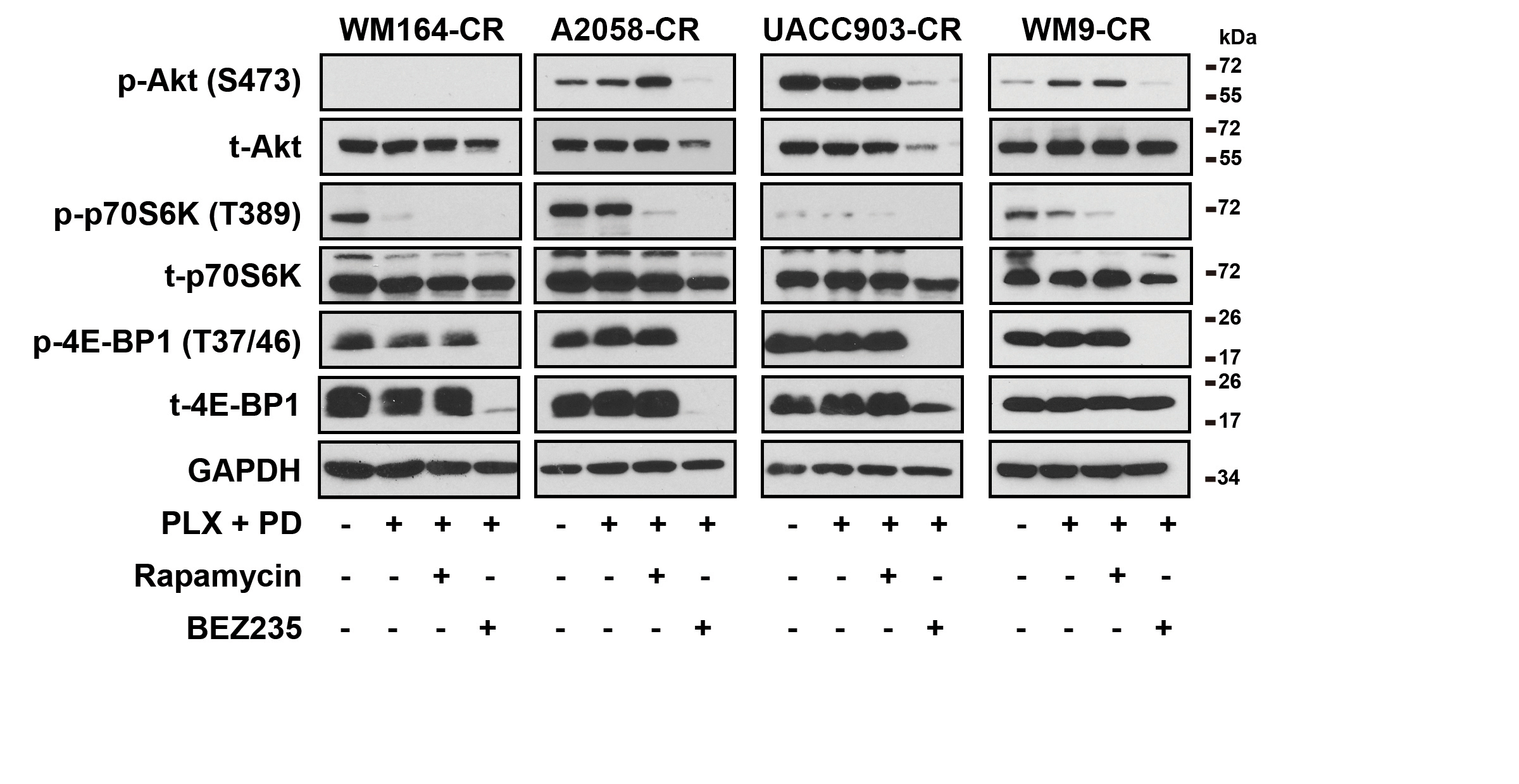

Supplement: Supplementary file 3 — Activation of AKT/mTOR signaling pathway in CR melanoma cells treated with Rapamycin or NVP-BEZ235. [file 41388_2021_1911_MOESM3_ESM.jpg]

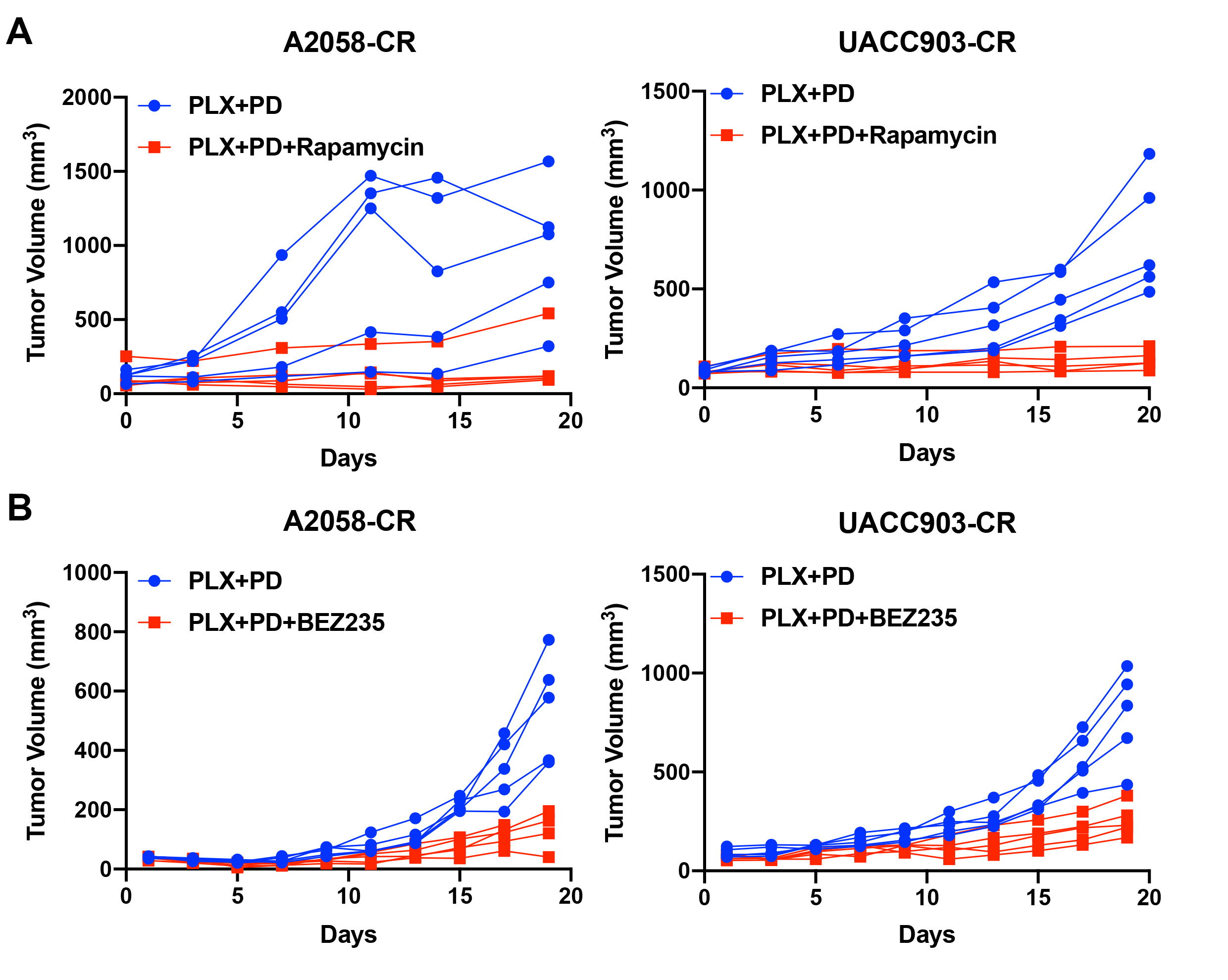

Supplement: Supplementary file 4 — Spider blots of the CR melanoma xenograft tumor growth when treated with rapamycin or NVP-BEZ235 in the presence of BRAF and MEK inhibitors. [file 41388_2021_1911_MOESM4_ESM.jpg]

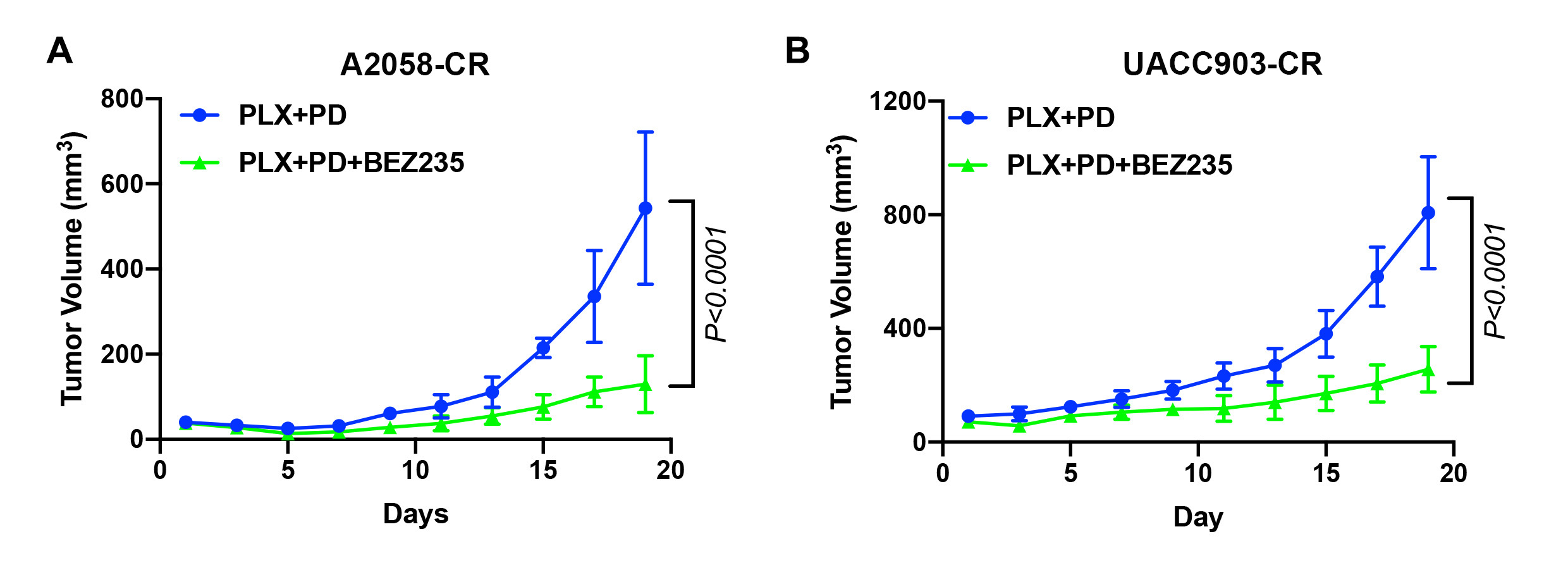

Supplement: Supplementary file 5 — NVP-BEZ235 suppresses the growth of CR melanoma tumors. [file 41388_2021_1911_MOESM5_ESM.jpg]
