# Supplementary material for: Targeting mTOR signaling overcomes acquired resistance to combined BRAF and MEK inhibition in BRAF-mutant melanoma
Source: Oncogene. 2021 Jul 24;40(37):5590–9. doi: 10.1038/s41388-021-01911-5 (PMC8445818; doi:10.1038/s41388-021-01911-5)
Supplement: Supplementary file 6 — Supplementary Table and Supplementary Figure Legends [file 41388_2021_1911_MOESM6_ESM.docx]

**SUPPLEMENTARY TABLES**

**Table S1. Information of antibodies used in the study**

| **Antibody** | **Provider** | **Application** | **Identifier** |
| --- | --- | --- | --- |
| Anti-phospho-Rb (S807/811)  Anti-phospho-ERK (T202/Y204) | Cell Signaling Technology  Cell Signaling Technology | WB  WB | #8516  #4370 |
| Anti-ERK | Cell Signaling Technology | WB | #9102 |
| Anti-phospho Akt (S473) | Cell Signaling Technology | WB | #9271 |
| Anti-Akt (pan) | Cell Signaling Technology | WB | #4691 |
| Anti-phospho-p70-S6K (T389) | Cell Signaling Technology | WB | #9205 |
| Anti-p70-S6K | Cell Signaling Technology | WB | #9202 |
| Anti-phospho-S6 (S240/244) | Cell Signaling Technology | WB; IHC | #2211 |
| Anti-S6 | R&D system | WB | MAB5436 |
| Anti-phospho-4E-BP1 (T37/46) | Cell Signaling Technology | WB | #2855 |
| Anti-4E-BP1 | Cell Signaling Technology | WB | #9452 |
| Anti-PTEN | Cell Signaling Technology | WB | #9188 |
| Anti-GAPDH | Cell Signaling Technology | WB | #2118 |
| Anti-Ki67 | Cell Signaling Technology | IHC | #9027 |
| Anti-cyclin B1 | Cell Signaling Technology | WB | #4135 |
| Anti-Bcl-xl | Cell Signaling Technology | WB | #2764 |
| Anti-cleaved-caspase3 (Asp175) | Cell Signaling Technology | WB; IHC | #9661 |

WB: Western blotting; IHC: Immunohistochemistry.

**Table S2. Pathway score predictors [87]**

| **TSC/mTOR score** | **Direction** | **PI3K/AKT score** | **Direction** |
| --- | --- | --- | --- |
| 4EBP1PS65 | + | AKTPS473 | + |
| 4EBP1PT37T46 | + | AKTPS308 | + |
| P70S6KPT389 | + | GSK3ALPHABETAPS21S9 | + |
| MTORPS2448 | + | P27PT157 | + |
| S6PS235S236 | + | P27PT198 | + |
| S6PS240S244 | + | PRAS40PT246 | + |
| RICTORPT1135 | + | TUBERINPT1462 | + |
| **ERK score** | **Direction** | INPP4B | - |
| MAPKPT202Y204 | + | PTEN | - |
| **Cell cycle score** | **Direction** | **Apoptosis score** | **Direction** |
| CDK1 | + | BAK | + |
| CYCLINB1 | + | BAX | + |
| CYCIND1 | + | BID | + |
| CYCLINE1 | + | BIM | + |
| P27PT157 | + | CASPASE7CLEAVED | + |
| P27PT198 | + | BADPS112 | - |
| PCNA | + | BCL2 | - |
|  |  | BCLXL | - |
|  |  | CIAP | - |

**SUPPLEMENTAL FIGURES:**

**Figure S1.** **Analysis of CR cell growth using MTT assay.**

(A, C, E, G) Relative survival of WM164-CR, A2058-CR, UACC903-CR, and WM9-CR cells treated with increasing concentrations of Rapamycin (Rapa, Max=100 µM), PLX-4720 (PLX, Max=100 µM) and PD0325901 (PD, Max=10 µM), and their combination with Rapamycin.

(B, D, F, H) Relative survival of WM164-CR, A2058-CR, UACC903-CR, and WM9-CR cells treated with increasing concentrations of NVP-BEZ235 (BEZ, Max=100 µM), PLX-4720 (PLX, Max=100 µM) and PD0325901 (PD, Max=10 µM), and combined NVP-BEZ235 with PLX-4720.

**Figure S2. mTOR inhibitors suppress the viability of CR cells *via* decreasing proliferation and inducing apoptosis.**

(A) Representative images of crystal violet assay for WM164-CR, A2058-CR, UACC903-CR and WM9-CR cells that were treated with Rapamycin or NVP-BEZ235 in the presence of PLX-4720 and PD0325901 for 3 days.
(B) Quantification of the crystal violet staining in A.

(C) EdU incorporation assays for detection of proliferating cells (green) of WM164-CR, UACC903-CR and A2058-CR melanoma cells treated with Rapamycin and NVP-BEZ235 in the presence of PLX-4720 and PD0325901. The nuclei were stained with DAPI (blue).

(D) Flow cytometry of CR melanoma cells with indicated treatment using Annexin V/7-AAD double staining assays.

Data are presented as mean ± SD (n=3). P-values are from a two-sided unpaired Student’s *t*-test.

**Figure S3. Activation of AKT/mTOR signaling pathway in CR melanoma cells treated with Rapamycin or NVP-BEZ235.**

Western blotting analysis of the phosphorylation levels of AKT/mTOR related proteins. All cell lines were treated with Rapa or BEZ in the presence of PLX-4720 and PD0325901 for 24 hr. Data are representative of 3 independent biological experiments.

**Figure S4. Spider blots of the CR melanoma xenograft tumor growth when treated with rapamycin or NVP-BEZ235 in the presence of BRAF and MEK inhibitors.**

**Figure S5. NVP-BEZ235 suppresses the growth of CR melanoma tumors.** Tumor growth curve of A2058-CR xenografts (A) and UACC903-CR xenografts (B) treated with vehicle or NVP-BEZ235 in the presence of PLX-4720 and PD0325901, 5 mice for each indicated group. Data are presented as mean ± SD. P-values are from two-way ANOVA assays.
